# ebGSEA: an improved Gene Set Enrichment Analysis method for Epigenome-Wide-Association Studies

**DOI:** 10.1093/bioinformatics/btz073

**Published:** 2019-01-31

**Authors:** Danyue Dong, Yuan Tian, Shijie C Zheng, Andrew E Teschendorff

**Affiliations:** 1 CAS Key Lab of Computational Biology, CAS-MPG Partner Institute for Computational Biology, Shanghai Institute of Nutrition and Health, Shanghai Institute for Biological Sciences, University of Chinese Academy of Sciences, Chinese Academy of Sciences, Shanghai, China; 2 UCL Cancer Institute, University College London, London, UK

## Abstract

**Motivation:**

The biological interpretation of differentially methylated sites derived from Epigenome-Wide-Association Studies (EWAS) remains a significant challenge. Gene Set Enrichment Analysis (GSEA) is a general tool to aid biological interpretation, yet its correct and unbiased implementation in the EWAS context is difficult due to the differential probe representation of Illumina Infinium DNA methylation beadchips.

**Results:**

We present a novel GSEA method, called ebGSEA, which ranks genes, not CpGs, according to the overall level of differential methylation, as assessed using all the probes mapping to the given gene. Applied on simulated and real EWAS data, we show how ebGSEA may exhibit higher sensitivity and specificity than the current state-of-the-art, whilst also avoiding differential probe representation bias. Thus, ebGSEA will be a useful additional tool to aid the interpretation of EWAS data.

**Availability and implementation:**

*ebGSEA* is available from https://github.com/aet21/ebGSEA, and has been incorporated into the ChAMP Bioconductor package (https://www.bioconductor.org).

**Supplementary information:**

Supplementary data are available at *Bioinformatics* online.

## 1 Introduction

The number of Epigenome-Wide-Association Studies (EWAS) has grown rapidly, yet the biological interpretation of the differentially methylated sites found in these studies remains a significant problem (Lappalainen and Greally, 2017; Teschendorff and Relton, 2018). EWAS typically use Illumina Infinium beadchips to measure DNA methylation (DNAm) at over 480 000 or 850 000 CpGs, depending on the beadchip version (Beck, 2010; Moran *et al.*, 2016), and genes represented on these chips may have widely different numbers of probes mapping to them (Phipson *et al.*, 2016). It has been noted that this differential probe representation may cause significant bias when conducting differential methylation and Gene Set Enrichment Analysis (GSEA), favouring genes with more probes (Geeleher *et al.*, 2013; Phipson *et al.*, 2016). This is similar to the well-known bias of RNA-Seq differential expression calls towards longer genes, and for this reason, methods that adjust for this bias in RNA-Seq data have been adapted to the DNAm context (Phipson *et al.*, 2016). However, drawing an analogy between RNA-Seq and DNAm data is also misleading, because in the RNA-Seq context the length of the gene only affects the reliability of the measured expression level, whereas in the DNAm context, the reliability of the measured DNAm level at a given CpG site does not depend on the number of probes mapping to the same gene. Thus, although genes with higher probe representation are more likely to be called differentially methylated, directly adapting methods from RNA-Seq data to DNAm beadchips may introduce other biases and still lead to suboptimal GSEA.

## 2 Description

**Fig. 1.**
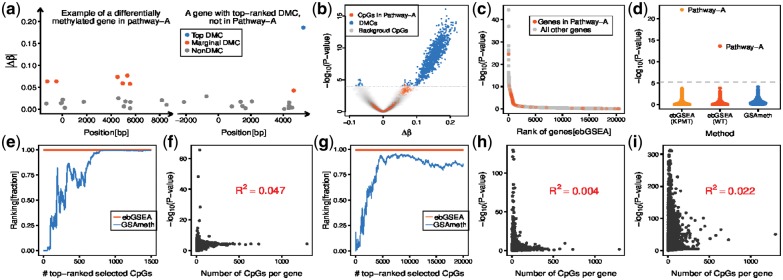
Validation of ebGSEA. (**a**) Example of a differentially methylated gene mapping to a hypothetical ‘pathway-A’, and of a gene containing a top-ranked DMC not mapping to pathway-A. *Y*-axis labels the absolute differential methylation between two phenotypes. Each datapoint corresponds to a CpG mapping to the gene, with the position relative to the transcription start site (*x*=0). (**b**) Volcano plot of the resulting DMCs with the grey dashed line indicating the line of significance (FDR=0.05). (**c**) Significance (*y*-axis) versus rank position of the gene (*x*-axis), as ranked by ebGSEA. (**d**) Significance of 8567 biological terms, as assessed using GSAmeth and ebGSEA combined with either a Wilcoxon test or the Known Population Median test. Dashed line marks Bonferroni threshold. (**e**) Plot of the rank position (expressed as a fraction) of a biological term containing genes overexpressed in smoking-related head&neck cancer in a smoking-EWAS performed in buccal swabs versus the number of top-ranked selected CpGs used in GSAmeth (blue line). Red line indicates the rank position of the same term under ebGSEA. (**f**) Significance of the genes, as given by ebGSEA, versus the number of CpGs mapping to the gene, as derived using ebGSEA in the same smoking-EWAS. *R*^2^ value demonstrates that ebGSEA is unbiased. (**g–h**) As (e–f), but now for a term of transcriptionally altered genes in an age-EWAS performed in blood. (**i**) As panels f and h, but now for rheumatoid arthritis in an EWAS performed in blood

Here we present a novel GSEA method for Illumina DNAm data, with an empirical Bayes interpretation (thus called ebGSEA), which overcomes the differential probe representation bias, whilst also avoiding some of the residual biases of current state-of-the-art methods like GSAmeth (Phipson *et al.*, 2016). GSAmeth works by ranking differentially methylated CpGs (DMCs), selecting those that pass a genome-wide significance threshold, then mapping these to genes and finally to biological terms (pathways). Adjustment for differential probe representation is carried out at the gene-mapping stage, whereby the significance of the number of DMCs mapping to a given gene is assessed in relation to how many probes map to that gene. This, however, may result in two undesirable outcomes. First, genes in a pathway where a substantial fraction of marginal DMCs do not pass genome-wide significance levels may result in the enrichment of the pathway being missed (Fig. 1a–d, Supplementary Material). Second, two pathways, matched for all variables (number of genes, probes mapping to each gene, number of genes containing at least one DMC) but differing widely in terms of the number or effect size of DMCs within a gene, will be ranked equally (Supplementary Material). ebGSEA overcomes these problems by adapting the global test (Goeman *et al.*, 2004) to directly rank genes according to their overall level of differential methylation, as assessed using all of the probes that map to the given gene and in a manner which avoids favouring genes containing more probes. Subsequently, enrichment of biological terms is performed on this ranked list of genes using either a standard one-tailed Wilcoxon rank sum test, or a recently introduced more powerful version known as the Known Population Median test (Parks, 2018). As a result, in the first scenario considered above, affected genes will be relatively highly ranked via ebGSEA (Fig. 1c), and the ensuing ranked list leads to significant enrichment of the pathway (Fig. 1d). In the second scenario, ebGSEA will favour the pathway containing more DMCs, as required (Supplementary Material).

We also compared ebGSEA to GSAmeth in a smoking-EWAS performed on 400 buccal swabs (Teschendorff *et al.*, 2015). Here, ebGSEA ranked a biological term associated with smoking-related head&neck cancer much more highly than GSAmeth, the latter exhibiting wide variation depending on the number of top-ranked DMCs (Fig. 1e, Supplementary Material). For instance, selecting the top-500 DMCs, GSAmeth would not have ranked this smoking-related term among the top-25% enriched ones, in contrast to ebGSEA which ranked it among the top 1% (Fig. 1e). Of note, the ranking or statistical significance of genes derived from ebGSEA did not correlate with the number of CpGs mapping to the gene, confirming that ebGSEA, like GSAmeth, avoids differential probe representation bias (Fig. 1f). Similar results were observed in other EWAS (Fig. 1g–i, Supplementary Material).

## 3 Conclusion

We propose that ebGSEA be used alongside other GSEA methods to obtain a more objective and comprehensive assessment of GSEA in a given EWAS.

## Funding

A.E.T. was supported by the Royal Society and Chinese Academy of Sciences [Newton Advanced Fellowship 164914]; and the National Natural Science Foundation of China [grant numbers: 31571359, 31771464, 31401120].

*Conflict of Interest:* none declared.

## Supplementary Material

btz073_Supplementary_Information
