# Supplementary material for: ebGSEA: an improved Gene Set Enrichment Analysis method for Epigenome-Wide-Association Studies
Source: Bioinformatics. 2019 Jan 31;35(18):3514–6. doi: 10.1093/bioinformatics/btz073 (PMC6748733; doi:10.1093/bioinformatics/btz073)
Supplement: btz073_Supplementary_Information [file btz073_supplementary_information.pdf]

Supplementary Information for *ebGSEA: An improved Gene Set Enrichment Analysis method for Epigenome-Wide Association Studies*

Extended Abstract

Gene Set Enrichment Analysis is one of the most common tasks in the analysis of omic data, and is critical for biological interpretation. In the context of Epigenome-Wide Association Studies, which typically rank individual cytosines according to the level of differential methylation, enrichment analysis of biological pathways is challenging due to differences in CpG/probe density between genes. While a number of recent methods, which overcome this bias have been proposed, we have found that these algorithms may overadjust, rendering them less powerful to detect genuine biological enrichment. Here we propose an empirical Bayes Gene Set Enrichment Analysis (ebGSEA) algorithm, which does not rank CpGs but genes according to the overall level of differential methylation of its CpGs/probes, allowing unbiased and sensitive detection of enriched pathways. Using simulated data and real data scenarios, we demonstrate that ebGSEA substantially outperforms the existing state-of-the-art. ebGSEA is freely available and anticipate that it will become the tool of choice for GSEA in the context of DNA methylation studies.

Background

The most common task in omic data analysis is the ranking of features (e.g. genes/CpGs/SNPs) in relation to some phenotype or factor of interest (e.g. case-control status, age, obesity). The main purpose of this ranking is to subsequently establish if specific biological pathways (or other biological terms) are enriched among the highly ranked features, which would indicate that these pathways or biological terms are altered, or associated, with the factor of interest. While in the context of gene expression, rankings are often derived at the level of genes, in the context of Epigenome-Wide Association Studies (EWAS), which measure DNA methylation (DNAm) at individual cytosines, rankings are most often derived at the level of individual cytosines, genomic regions, or probes, depending on the underlying technology. However, a well-known problem is that the number of cytosines or probes can vary dramatically between genes, which may lead to genes with higher CpG or probe density having an intrinsically

higher probability of being highly ranked. Thus, if genes appearing in specific biological terms have abnormally higher CpG or probe density, not adjusting for this bias could lead to false enrichment [1, 2].

A similar bias may also occur in the context of RNA-Seq data, where longer genes are intrinsically more likely to exhibit differential expression [3], although in this context the bias is caused by the fact that expression levels are measured more reliably for longer genes. An algorithm called *goseq* was proposed to avoid this bias in the context of RNA-Seq data [3], and the underlying method was recently adapted to tackle the differential probe representation bias of DNA methylation data generated with Illumina Infinium beadarrays [1]. This method is called GSAmeth and is part of the missMethyl Bioconductor package. While GSAmeth is efficient at removing this bias, we have found that this algorithm may overadjust, rendering the method less sensitive to detect genuine biological enrichment. Here, we explicitly demonstrate this using two different real EWAS, where specific biological terms should be enriched and where GSAmeth does not predict their enrichment. In addition, we also find that GSAmeth may introduce other biases: for instance, it does not care about the number of DMCs that map to a gene, nor about their significance levels, all of which can lead to suboptimal ranking of enriched biological terms, and thus to a potentially reduced sensitivity and specificity.

To address these issues, we propose a novel empirical Bayes algorithm (called ebGSEA), which unlike most other methods, ranks genes instead of CpGs/probes. ebGSEA leverages the evidence of differential methylation from all CpGs/probes mapping to a given gene, to rank genes according to their overall level of differential methylation. A key property of ebGSEA is that, like GSAmeth, it does not favour genes with high or low CpG/probe representation, thus avoiding the bias, whilst also rendering the method sensitive enough to detect true biological enrichment. With genes ranked by this empirical Bayes regression model, GSEA can subsequently be performed using a non-parametric Wilcoxon rank sum test or the known-population median test (KPMT) [4], thus allowing GSEA to be performed in a threshold independent manner.

The ebGSEA algorithm

As mentioned, ebGSEA has two direct advantages over a competing method like GSAmeth. First, it directly ranks genes according to their overall level of differential methylation, as assessed using all of the probes that map to the given gene, without incurring differential probe representation bias. Second, because ebGSEA ranks genes, enrichment of biological terms can be performed on this ranked list using either a standard one-tailed Wilcoxon rank sum test (WT), or a recently introduced more

powerful version called Known Population Median Test (KPMT) [4], thus avoiding the need for what is normally an arbitrary choice of threshold for calling significant genes. This is in contrast to GSAmeth, which ranks probes and which subsequently requires the specification of a significance threshold to declare a list of significant DMCs.

ebGSEA ranks genes according to their level of differential methylation by adapting the *global test* from Goeman et al [5-7], which can be interpreted either as a random effects model, or alternatively, as an empirical Bayes generalized regression model. Specifically, the *global-test* evaluates whether the DNA methylation patterns of CpGs mapping to a given gene *g* differ significantly between two phenotypes. Assuming that the phenotype of samples labeled by an index *s* is encoded in a vector *Y,* then the model for gene *g* is

$$E_g[Y_s|\gamma_g] = h^{-1}(\alpha_g + \sum_{c=1}^{n_{cg}} \gamma_{cg} m_{cs})$$

where h is the link function, α_g is the intercept term, m_{cs} is the methylation (beta-value) for CpG *c* (that maps to gene *g*) in sample *s*, γ_{cg} are regression coefficients to be estimated, and where n_{cg} is the total number of probes (CpGs) mapping to gene *g*. Since this number could be large, testing the null that all $\gamma_{cg} = 0$ can be conveniently formulated in an empirical Bayesian setting where one assumes that all γ_{cg} are drawn from a common distribution of mean zero and variance σ^2. The null hypothesis then becomes $\sigma^2 = 0$. The observed methylation data of the CpGs mapping to the gene is then used to determine the posterior probability that $\sigma^2 > 0$. As shown by Goeman et al, and interpreting the above model as a random effects model, a score test can be constructed which is locally most powerful on average in a neighborhood of the null hypothesis [8]. The test is therefore specially powerful for detecting alternatives characterized by many weak effects (e.g. many marginal DMCs mapping to the same gene). The test yields a P-value, that allows all genes to be ranked based on the combined evidence for differential methylation of its constituent probes.

ebGSEA improves ranking and sensitivity on simulated data

To illustrate some of the key advantages of ebGSEA, we considered specific simulation models. In order to avoid the influence of gene overlaps between different pathways, we devised a simulation framework whereby altered "pathway(s) of interest" were constructed by selecting genes with representative probes on the Illumina beadarrays (450k or EPIC), but which were not found in the Molecular Signatures Database (MsigDB) [9] (the database we use to perform GSEA) (**Fig.S1**). In other words, we use a strategy whereby the 8567 biological terms of MSigDB contain genes that are not

altered in relation to the phenotype of interest. These define our "true negative pathways" allowing us to more reliably estimate the specificity. In order to estimate sensitivity, we augment the MSigDB with new hypothetical altered pathways (the true positives) consisting of genes with probe representation on the beadarrays but which are not found in the original MSigDB database. These genes are allowed to contain DMCs, as specified in more detail below. The lack of gene overlap between our altered pathways of interest and all those in the MSigDB database allows us to assess the specificity in an unbiased way. In more detail, we considered the following simulation models:

Simulation model-1: We defined two altered pathways of interest (A & B), as described above, matched for all variables (i.e. number of genes in pathway, probes mapping to each gene and number of genes containing at least 1 DMC). In pathway A, all CpGs mapping to a differentially methylated gene (DMG) are DMCs (we model these from a different beta distribution so that the average difference in DNAm is large, $\Delta\beta = 0.6$). In pathway B, only one CpG mapping to an altered gene is a DMC. Thus, in this scenario both pathways have the same number of DMGs (as the DMCs occur at very high statistical significance), but the number of DMCs within a DMG are wildly different. **Fig.S2a-b** depicts this scenario. As results show, GSAmeth assigns the same P-value to those two pathways (**Fig.S2c**), whereas ebGSEA favors the pathway containing more DMCs, as required. The inability of GSAmeth to properly rank pathways occurs because it first ranks DMCs, then maps DMCs to genes, assigning same weight to genes with lots of DMCs than to genes with only one DMC.

Simulation Model-2: In this scenario we again consider two pathways (A & B), but in this case all the CpGs mapping to DMGs are DMCs. The difference between A and B is in terms of the level of statistical significance of the DMCs, with DMCs in pathway A exhibiting high statistical significance ($\triangle\beta = 0.6$), whereas DMCs in pathway B exhibit more marginal differences in DNAm ($\triangle\beta = 0.2$). Thus, although the number of DMCs and DMGs are the same in the two pathways, the significance levels of the two pathways should be different, since for pathway-A, the associated effect sizes are much bigger. **Fig.S3a-b** depicts this scenario, and as we can see, GSAmeth assigns almost the same P-value to these two pathways, whereas ebGSEA favors the pathway containing the more significant DMCs (**Fig.S3c**), as required.

Simulation Model-3: We now only have one altered pathway "A" containing 50 genes with 25% of the CpGs mapping to them exhibiting marginal DNAm changes ($\triangle\beta = 0.15$). These marginal DMCs do not pass genome-wide significance levels. We also randomly choose 1000 CpGs from the full background set of 450k CpGs to be DMCs ($\triangle\beta = 0.3$), that do pass genome-wide significance levels. We refer to these as background DMCs as these are randomly chosen and therefore not necessarily associated with any pathway. **Fig.1a** depicts an example of a gene in pathway A, and

of another gene not in pathway-A but containing a top ranked DMC. We can see that genes in pathway-A contain a lot of marginal DMCs which will not be selected as DMCs in GSAmeth (**Fig.1b**), resulting in the enrichment of the pathway being missed by GSAmeth. In contrast, those genes will be relatively highly ranked via ebGSEA, and the ensuing ranked list leads to significant enrichment of the pathway (**Fig.1c-d**).

Simulation Model-4: We implemented Simulation Model-3 more systematically in order to better estimate sensitivity and to assess its dependence on the number of background DMCs. We allowed the number of background DMCs to vary from 1000, 3000 to 5000. We also used two different thresholds to call significance at the pathway level: (1) Bonferroni-corrected, i.e. using 0.05/(number of pathways) as the P-value threshold, and (2) using Benjamini-Hochberg FDR and selecting those with FDR<0.05. We also considered the two different versions of beadarrays: EPIC [10] and Illumina 450k [11]. We ran a total of 100 Monte-Carlo simulations to obtain an average sensitivity and specificity for each method (ebGSEA/GSAmeth). Results confirm our previous analysis, in that ebGSEA can achieve very high sensitivity in scenarios where GSAmeth would not identify the truly altered pathway of interest, and that this increased sensitivity does not occur at the expense of a substantially decreased specificity (**Fig.S4**). We also modified Model-4 to include 50 altered pathways of interest, instead of only 1. Each of the 50 altered pathways consists of 50 genes, thus making a total of 250 genes, all selected from genes represented on the beadarray but not part of the MSigDB database. As before, 25% of the CpGs mapping to genes in each of the 50 pathways were chosen to be altered at a marginal level ($\triangle\beta$ =0.15). Results for sensitivity are in line with those obtained earlier (**Fig.S5**).

ebGSEA avoids differential probe representation bias

We further evaluated ebGSEA on 3 independent real EWAS HM450k datasets.

First is a buccal swab dataset [12]. We here use the discovery dataset which contains 400 buccal swab samples from women all aged 53 at sample draw and who varied significantly in terms of their smoking exposure. As shown previously using Fisher's exact GSEA method, a biological term containing genes overexpressed in smoking related head & neck cancer was highly enriched among smoking-associated DMCs derived from these buccal swabs [12]. This makes biological sense since the epithelial cells from the buccal swabs are likely to serve as close proxies for the cell of origin of specific head & neck cancers (nasopharyngeal carcinoma). Thus, we can objectively test ebGSEA and GSAmeth in their ability to detect enrichment of this specific

biological term. The biological term in question is DODD_NASOPHARYNGEAL_CARCINOMA_UP and is part of MSigDB [9]. To derive smoking DMCs, we followed the procedure in [12], performing linear regressions between smoking pack years and DNAm using bisulfite conversion efficiency as a covariate.

The second dataset is one of the largest available EWAS for aging [13]. We downloaded this Illumina 450k data from GEO (GSE40279). This dataset contains 656 whole blood samples. We used Singular Value Decomposition (SVD) to asses the sources of inter-sample variation. This showed that variation of this dataset was mostly driven by the source site of samples, plate and ethnicity, with gender and age associated with lower ranked components. Since source and ethnicity were fully correlated with plate, here we used *"Combat"* function in *sva* R-package to remove the plate effect. Age was used as the phenotype to subsequently identify DMCs. As a gold-standard biological term we used the list of genes differentially expressed in peripheral blood as a function of age from Peters et al [14]. These age-associated mRNA changes co-locate with potentially functional CpG methylation sites in enhancer and insulator regions, and are thus likely to be accompanied by DNAm changes, providing a non-trivial but also objective test.

The third dataset is a Rheumatoid arthritis (RA) EWAS study by Liu et al [15]. It is an Illumina 450k dataset of 689 peripheral blood samples, which we downloaded from GEO (GSE42861). In this set we used RA as the phenotype, and because RA is known to be associated with a shift in the granulocyte to lymphocyte ratio, we would expect biological terms related to the immune system to be highly enriched among top-ranked DMCs associated with RA. We note that DMCs were derived not adjusting for blood cell subtype fractions, since we want to test whether the GSEA method can capture the shift in the granulocyte to lymphocyte ratio.

We checked that in all 3 datasets, ebGSEA avoids differential probe representation bias, since the statistical significance of the DMGs did not correlate with the number of CpGs mapping to the gene (**Fig.S6, or Figs.1f,h,i**).

ebGSEA retains high sensitivity in real EWAS

To compare the sensitivity of ebGSEA to GSAmeth, we observed that ebGSEA ranked the corresponding gold-standard biological terms in each dataset very highly, whereas GSAmeth exhibited substantial variation as a function of the number of selected DMCs (**Fig.S7, Figs.1g,e**) (with the exception of the Liu et al set where both methods did well).

Implementation and availability

ebGSEA is available in ChAMP as function champ.ebGSEA().We can use the codes as below:

```
myLoad <-champ.load(directory=system.file("extdata",package="ChAMPdata"))
myNorm <- champ.norm()
myebGSEA<-
champ.ebGSEA(beta=myNorm,pheno=myLoad$pd$Sample_Group,arraytype="450K")
```

Where parameter beta is a matrix of values representing the methylation scores for each sample, pheno is the phenotype information.

Alternatively stand-alone functions are available from *http://github.com/ebGSEA/aet21*

Conclusions

In summary, ebGSEA, like GSAmeth, successfully avoids the bias associated with differential probe representation, whilst also allowing biological terms to be ranked depending on the number and statistical significance level of the DMCs present in the differentially methylated genes. The fact that ebGSEA ranks genes, not CpGs, is a succinct advantage, as it combines the information pertaining to the number of DMCs and their individual significance levels for each gene, to rank them in an unbiased fashion. Thus, ebGSEA also naturally avoids having to assign what is often an arbitrary statistical significance threshold for calling DMCs, since enrichment analysis methods based on ranked lists of genes can be used to obtain final rankings of enriched biological terms. Although we have demonstrated the validity of ebGSEA mainly on the HM450k platform, our simulation results on the EPIC beadarray suggest that its performance is largely independent of the version of Illumina beadarray platform. Thus, ebGSEA will be a useful GSEA tool for all upcoming EWAS that use EPIC beadarrays and for re-analysis of existing HM450k data.

Supplementary Figures

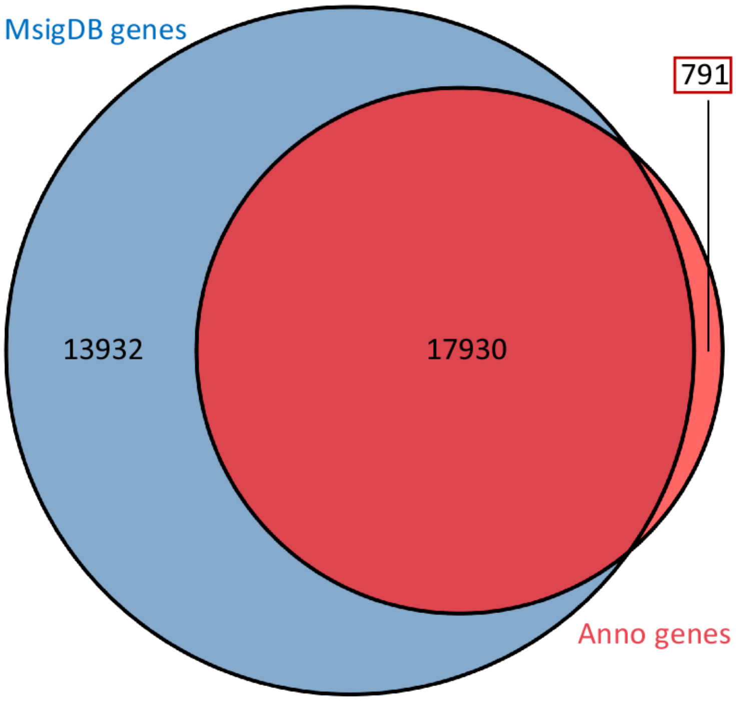

Fig.S1. Venn Diagram of genes in the MSigDB database and genes annotated to the HM450k beadarray. In MSigDB we have 31,862 unique Entrez Gene IDs, of which 17930 overlap with Entrez Gene IDs annotated to the HM450k platform. In total, we observed 719 genes that have no overlap with genes in MsigDB, and it is from this subset of 719 genes that we define new hypothetical pathways of interest (POI) which contain DMCs and are thus altered (defining true positives). All other pathways/biological terms in MSigDB contain no genes with DMCs and thus constitute true negatives. This strategy allows more reliable assessment of the specificity of GSEA methods.

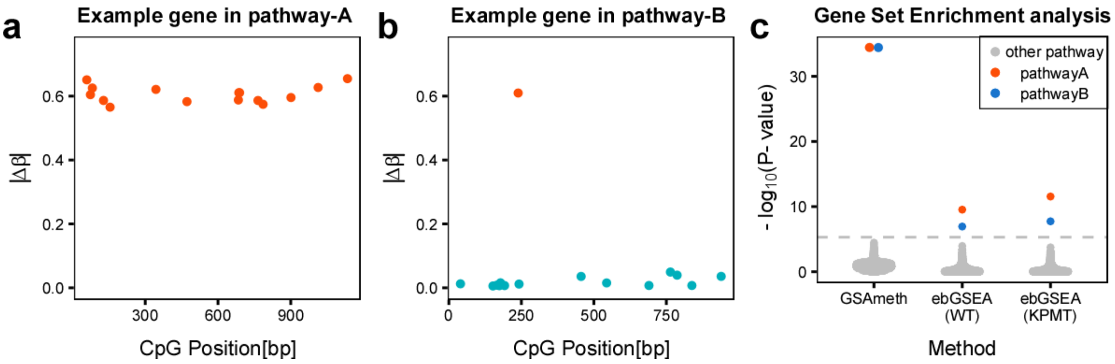

Fig.S2: **Specification and result of simulation model-1. a)** Example of a gene in pathway-A,: all the CpGs mapping to the gene exhibit highly significant and fairly large (~0.6) DNAm changes**. b)** Example of a gene in pathway-B: only one of the CpGs mapping to the gene is a DMC with a ~0.6 DNAm change. **c)** Pathway significance values (-log10(P), y-axis) for 8569 pathway terms and three different GSEA methods (x-axis). Red dot indicates pathway-A, blue dot indicates pathway-B. Grey dashed line indicates Bonferroni significance level. Observe that although gsameth assigns both pathways higher statistical significance levels than ebGSEA, that the statistical significance values from ebGSEA still pass a stringent Bonferroni-level and that it ranks pathway-A above pathway-B, as required.

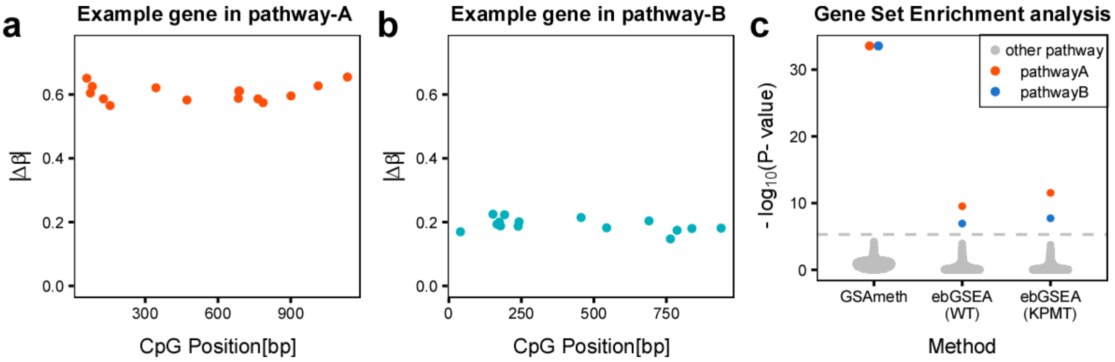

Fig.S3: **Specification and result of simulation model-2. a-c)** As in previous figure, but now with the CpGs mapping to genes in pathway-B all being marginal DMCs, in contrast to those in pathway-A where they are all highly significant DMCs. Again, observe that although gsameth assigns both pathways higher statistical significance levels than ebGSEA, that the statistical significance values from ebGSEA still pass a stringent Bonferroni-level and that it ranks pathway-A above pathway-B, as required.

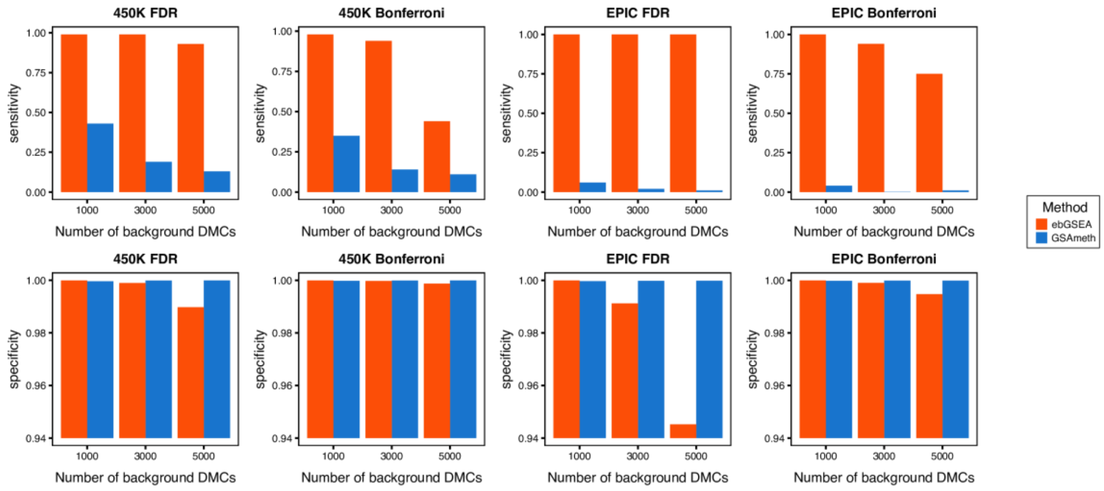

Fig.S4: **Result of simulation model-4 for the case of 1 altered pathway of interest. Top-row:** The average sensitivity of ebGSEA (orange) and GSAmeth (blue) to detect the 1 altered pathway of interest for 3 different numbers of background DMCs, for two different pathway significance thresholds (FDR<0.05 and Bonferroni) and for both HM450k and EPIC beadarray versions. **Bottom-row:** As top-row, but for the specificity.

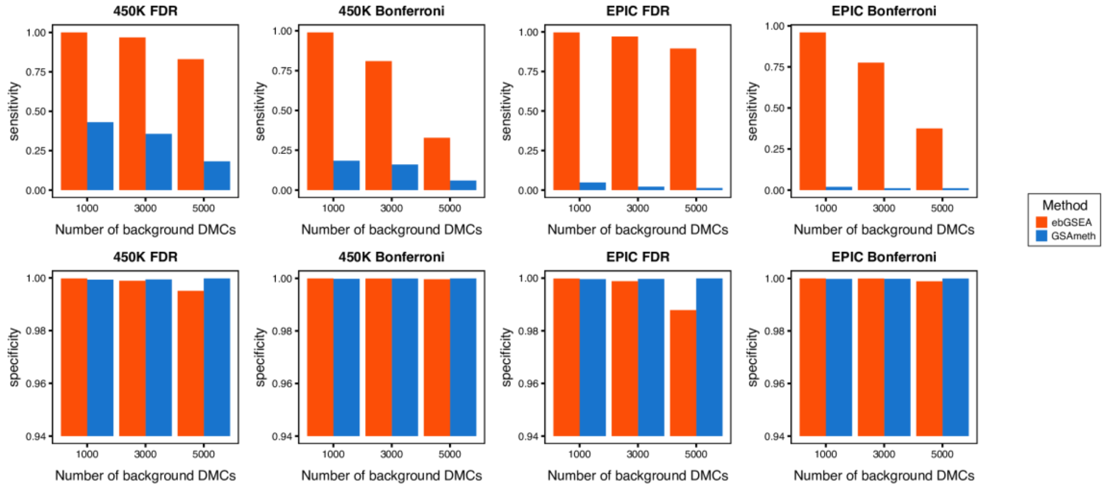

Fig.S5: **Result of simulation model-4 for the case of 50 altered pathways of interest. Top-row:** The average sensitivity of ebGSEA (orange) and GSAmeth (blue) to detect the 50 altered pathways of interest for 3 different numbers of background DMCs, for two different pathway significance thresholds (FDR<0.05 and Bonferroni) and for both HM450k and EPIC beadarray versions. **Bottom-row:** As top-row, but for the specificity.

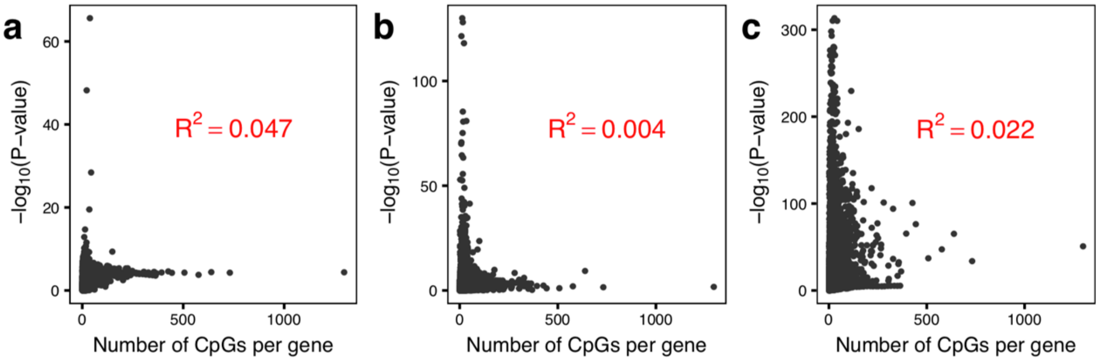

Fig.S6. ebGSEA avoids differential probe representation bias. Scatterplots of gene-level –$\log_{10}$(P-values) from the global-test used in ebGSEA against the number of CpGs per gene. We give the R^2 value, indicating absence of a correlation. **a)** Buccal Dataset, **b)** Hannum et al.'s dataset, **c)** Liu et al.'s dataset.

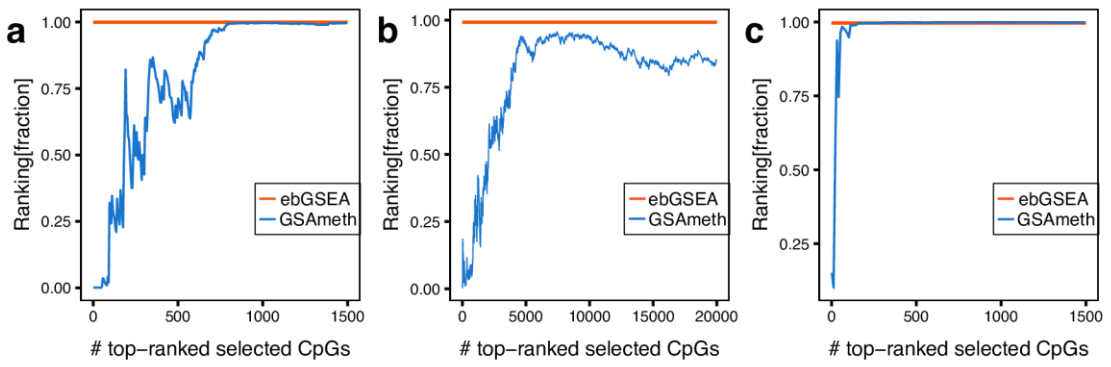

Fig.S7. ebGSEA retains high sensitivity in real EWAS. Ranking position (expressed as a fraction of all pathway terms) (y-axis) of a truly altered pathway against number of top-ranked selected CpGs (x-axis) for ebGSEA and GSAmeth. Ranking fraction is defined as Ranking fraction = 1 – (rank-1)/n, where n is the number of pathway terms and where rank is the rank position of the truly altered pathway. Thus, if pathway is ranked at the top, rank=1, and Ranking fraction is also 1. **a)** smoking-EWAS buccal set with smoking associated pathway ("DODD_NASOPHARYNGEAL_CARCINOMA_UP") as the truly altered pathway, **b)** Hanuum et al.'s whole blood EWAS for aging, with a truly altered pathway containing genes that are differentially expressed with chronological age in peripheral blood tissue. **c)** Liu et al.'s EWAS dataset (GSE42861), with immune associated pathway ("IMMUNE_SYSTEM_PROCESS") as the truly altered pathway.

REFERENCES

1. Phipson B, Maksimovic J, Oshlack A: **missMethyl: an R package for analyzing data from Illumina's HumanMethylation450 platform.** *Bioinformatics* 2016, **32:**286-288.
2. Geeleher P, Hartnett L, Egan LJ, Golden A, Raja Ali RA, Seoighe C: **Gene-set analysis is severely biased when applied to genome-wide methylation data.** *Bioinformatics* 2013, **29:**1851-1857.
3. Young MD, Wakefield MJ, Smyth GK, Oshlack A: **Gene ontology analysis for RNA-seq: accounting for selection bias.** *Genome Biol* 2010, **11:**R14.
4. Parks MM: **An exact test for comparing a fixed quantitative property**

between gene sets. *Bioinformatics* 2018, **34:**971-977.

5. Meijer RJ, Goeman JJ: **Multiple Testing of Gene Sets from Gene Ontology: Possibilities and Pitfalls.** *Brief Bioinform* 2016, **17:**808-818.

6. Chaturvedi N, Goeman JJ, Boer JM, van Wieringen WN, de Menezes RX: **A test for comparing two groups of samples when analyzing multiple omics profiles.** *BMC Bioinformatics* 2014, **15:**236.

7. Goeman JJ, van de Geer SA, de Kort F, van Houwelingen HC: **A global test for groups of genes: testing association with a clinical outcome.** *Bioinformatics* 2004, **20:**93-99.

8. Goeman JJ, le Cessie S: **A goodness-of-fit test for multinomial logistic regression.** *Biometrics* 2006, **62:**980-985.

9. Subramanian A, Tamayo P, Mootha VK, Mukherjee S, Ebert BL, Gillette MA, Paulovich A, Pomeroy SL, Golub TR, Lander ES, Mesirov JP: **Gene set enrichment analysis: a knowledge-based approach for interpreting genome-wide expression profiles.** *Proc Natl Acad Sci U S A* 2005, **102:**15545-15550.

10. Moran S, Arribas C, Esteller M: **Validation of a DNA methylation microarray for 850,000 CpG sites of the human genome enriched in enhancer sequences.** *Epigenomics* 2016, **8:**389-399.

11. Sandoval J, Heyn H, Moran S, Serra-Musach J, Pujana MA, Bibikova M, Esteller M: **Validation of a DNA methylation microarray for 450,000 CpG sites in the human genome.** *Epigenetics* 2011, **6:**692-702.

12. Teschendorff AE, Yang Z, Wong A, Pipinikas CP, Jiao Y, Jones A, Anjum S, Hardy R, Salvesen HB, Thirlwell C, et al: **Correlation of Smoking-Associated DNA Methylation Changes in Buccal Cells With DNA Methylation Changes in Epithelial Cancer.** *JAMA Oncol* 2015, **1:**476-485.

13. Hannum G, Guinney J, Zhao L, Zhang L, Hughes G, Sadda S, Klotzle B, Bibikova M, Fan JB, Gao Y, et al: **Genome-wide methylation profiles reveal quantitative views of human aging rates.** *Mol Cell* 2013, **49:**359-367.

14. Peters MJ, Joehanes R, Pilling LC, Schurmann C, Conneely KN, Powell J, Reinmaa E, Sutphin GL, Zhernakova A, Schramm K, et al: **The transcriptional landscape of age in human peripheral blood.** *Nat Commun* 2015, **6:**8570.

15. Liu Y, Aryee MJ, Padyukov L, Fallin MD, Hesselberg E, Runarsson A, Reinius L, Acevedo N, Taub M, Ronninger M, et al: **Epigenome-wide association data implicate DNA methylation as an intermediary of genetic risk in rheumatoid arthritis.** *Nat Biotechnol* 2013, **31:**142-147.
